# Editorial: Pathophysiology, treatment and rehabilitation of atherosclerosis-related diseases in geriatric population

**DOI:** 10.3389/fmed.2024.1358769

**Published:** 2024-02-08

**Authors:** Yan Xu, Xianwei Zeng, Wen-Jun Tu

**Affiliations:** ^1^Department of Neurosurgery, Beijing Tiantan Hospital, Capital Medical University, Beijing, China; ^2^Department of Neurosurgery, Rehabilitation Hospital of the National Research Center for Rehabilitation Technical Aids, Beijing, China; ^3^Geriatrics Innovation Center, Weifang People's Hospital, Weifang, China

**Keywords:** pathophysiology, treatment, rehabilitation, atherosclerosis, stroke

Atherosclerosis, a chronic cardiovascular ailment, significantly impacts human health and is a primary cause of death globally (1). Recognized as an age-related disease due to aging being a key risk factor, atherosclerosis often starts without symptoms but gradually leads to acute incidents such as heart attacks or strokes (2, 3). This slow progression poses a significant challenge for contemporary medicine. The increasing global impact of atherosclerosis, driven by an aging population, high prevalence, and suboptimal control of contributing factors like hypertension and diabetes, is concerning (4–6). Our Research Topic delves into the latest developments in understanding the origins, mechanisms, prevention, treatment, and management of atherosclerosis and its related conditions.

Atherosclerosis arises from a combination of genetic and environmental factors. For about a century, cholesterol, particularly the accumulation and alteration of low-density lipoprotein cholesterol (LDL-C) beneath the arterial endothelium, has been acknowledged as a significant contributor to its development (7). The elevation of LDL-C levels in the bloodstream leads to its deposition in the arterial walls of the heart and brain. This process gradually results in the formation of atherosclerotic plaques, which can obstruct blood vessels and potentially lead to severe conditions like coronary heart disease, stroke, and peripheral artery disease, all of which can cause death or disability (8). While the molecular mechanisms of atherosclerosis have been extensively explored through transgenic animal models both domestically and internationally, its exact pathogenesis remains elusive. Consequently, there's a need for new animal models and treatment methods, particularly for patients who do not adequately respond to statin therapy.

The discovery of immune cells within atherosclerotic plaques has led to extensive research into the role of inflammation in the disease's progression. Increasingly, atherosclerosis is being recognized as a chronic inflammatory and autoimmune condition (9). The foundational concept of this disease was first proposed in Russell Ross's “response-to-injury” hypothesis, which describes plaque growth and smooth muscle cell phenotype changes in relation to platelet-derived growth factors (10).

The process of atherosclerosis is intricate, involving various stages like local lipid deposition, LDL-C oxidation, endothelial dysfunction, vascular inflammation, cell adhesion, fibrosis, and the formation of atherosclerotic plaques (11). Initial pathophysiological changes predominantly include subendothelial lipid accumulation, endothelial malfunction, and the recruitment and clustering of monocytes (12).

At sites of vascular injury, the exposure of the subendothelial space to blood triggers platelet aggregation and the release of growth factors. Oxidation-modified LDL-C prompts monocytes to transform into macrophages, which secrete pro-inflammatory factors. These factors guide the migration and proliferation of smooth muscle cells from the media to the intima. These cells surround the lipid-rich necrotic foam cells and transition from a contractile to a synthetic phenotype. Stimulated by growth factors, they produce collagen and elastic fibers that form the extracellular matrix. Fibrous caps develop around the lipid pool, culminating in the formation of mature atherosclerotic plaques.

Acknowledging atherosclerosis as an active inflammatory condition, not merely passive cholesterol accumulation, has brought to light critical inflammatory mechanisms at play. The inflammation in atherosclerosis is driven by factors such as proinflammatory cytokines, inflammatory signaling pathways, bioactive lipids, and adhesion molecules. This inflammatory process forms a fundamental basis for both the physiological and pathological shifts that occur during the development and progression of atherosclerosis, playing a pivotal role at every stage of the atherogenic process (13).

The exploration into the roles of both the innate and adaptive immune systems in atherosclerosis has led to a more nuanced understanding of lesion development and has opened new pathways for research into treating this condition. Recently, several clinical studies have been conducted to assess the effectiveness of anti-inflammatory interventions in reducing cardiovascular disease risk. This approach serves as an alternative to traditional methods of managing classical risk factors (14, 15).

Atherosclerosis underpins the pathology of various cardiovascular diseases (16). The rupture of unstable atherosclerotic plaques, coupled with the narrowing or blockage of blood vessels, triggers platelet aggregation, and thrombosis. This sequence of events often precipitates acute cardiovascular incidents. The spectrum of diseases attributable to atherosclerosis encompasses coronary artery disease (CAD), lower extremity peripheral artery disease (PAD), and cerebrovascular disease. Gaining a deeper understanding of the pathogenesis of these conditions is crucial for the development of new therapeutic approaches and the identification of prognostic markers.

While high cholesterol levels are a primary risk factor for the development of atherosclerosis, and lipid-lowering medications such as statins are the cornerstone of treatment, there remains a residual cardiovascular risk in some individuals. This underscores the necessity for additional therapeutic measures. Recent research has revealed promising new targets for the treatment and prevention of atherosclerosis. Huang et al. discovered that circulating lipoprotein(a) [Lp(a)] molecules can directly deposit onto arterial walls, fostering foam cell formation and thus contributing to atherosclerosis. Notably, Lp(a) may play a causal role in the risk of various stroke types, including ischemic stroke and large artery atherosclerotic stroke (17, 18). This finding positions Lp(a) as a novel prognostic marker for atherosclerosis, potentially guiding future therapeutic strategies.

The primary cause of middle cerebellar peduncle (MCP) infarction is atherosclerotic stenosis or occlusion of the vertebrobasilar arteries. The MCP is notably the most frequent site of hearing loss in acute ischemic stroke. In middle-aged and elderly patients who have vascular risk factors and bilateral hearing loss, it's vital to routinely investigate vertebrobasilar artery disease as a secondary consequence of atherosclerosis. Yuan et al. emphasized the importance of thorough audiological and neurological assessments to pinpoint the exact lesion sites associated with hearing loss, which is crucial for timely intervention and rehabilitation.

In the context of osteoporotic fractures following stroke due to cerebral atherosclerosis, Li et al. identified oxidative stress as a key mechanism driving osteoporosis. Addressing oxidative stress-related pathways in the progression of cerebral atherosclerosis or mitigating ischemia-reperfusion injury could be instrumental in preventing post-stroke osteoporosis. This study offers insights into the pathogenesis and treatment options for stroke-related osteoporosis (Li et al.).

Concerning single subcortical infarction (SSI), which is often linked to atherosclerotic obstruction of parental arteries and significantly impacts prognosis, Yang et al. found that assessing parental arterial disease (PAD) and asymptomatic intracranial atherosclerotic stenosis (aIAS) through time-of-flight magnetic resonance angiography (MRA) is valuable in predicting adverse outcomes in SSI, particularly in patients without PAD. Evaluating aIAS may aid in stratifying the risk of post-stroke disability in SSI patients and guiding personalized care.

Acute coronary syndrome (ACS), resulting from the rupture of atherosclerotic plaques in the coronary artery, is a leading cause of death and morbidity in developed countries. Lectin like oxidized low-density lipoprotein receptor-1 (LOX-1) plays a significant role in the progression of atherosclerosis, as it is the primary endothelial cell receptor of oxidized low-density lipoprotein and is highly expressed in atherosclerotic lesions (19). Studies have shown that soluble LOX-1 levels are markedly increased in patients with ACS or coronary artery disease (CAD) and correlate with atherosclerotic plaque instability. This makes LOX-1 a potential prognostic marker for disease progression and the risk of future cardiovascular events (20).

Peripheral artery disease (PAD), caused by atherosclerosis, primarily affects the lower extremities and is characterized by symptoms like pain, intermittent claudication (IC), or critical limb ischemia (CLI). Patients with PAD often have concurrent CAD, placing them at an elevated risk of cardiovascular events. Treatment aims to mitigate this risk while enhancing exercise capacity and daily functionality. Current therapeutic approaches include the use of statins, antiplatelet agents, angiotensin-converting enzyme inhibitors (ACEIs), smoking cessation, and possibly antihypertensive medications (21).

Rehabilitation is crucial in enhancing the prognosis of atherosclerotic diseases and diminishing the risk of cardiovascular events. Cardiac rehabilitation, focusing on the comprehensive improvement and maintenance of cardiovascular health, is vital in improving the prognosis of coronary atherosclerotic heart disease (22). It involves personalized rehabilitation strategies aimed at achieving optimal physical, psychological, and social wellbeing. Percutaneous coronary intervention (PCI) is a common and effective clinical treatment for coronary atherosclerosis. While PCI can restore coronary circulation and augment myocardial perfusion, it cannot reverse the damage to necrotic heart cells. Therefore, cardiac rehabilitation post-PCI is essential. Research indicates that goal-oriented cardiac rehabilitation management can significantly enhance the postoperative self-management capabilities of patients with coronary atherosclerosis. It improves cardiac functions, exercise capacity, and effectively reduces the risk of cardiovascular events. In the context of peripheral artery disease (PAD), rehabilitation, including supervised exercise training and therapeutic education, is recommended as the primary treatment. Studies have shown that rehabilitation training notably improves pain-free walking distance, functional status, quality of life, and reduces cardiovascular risk factors and mortality in PAD patients (23).

For stroke patients resulting from cerebral atherosclerosis, rehabilitation services are key to promoting functional recovery and independence. Active rehabilitation effectively ameliorates movement and sensory disorders, communication difficulties, and overall quality of life (24, 25). However, the management of atherosclerotic disease rehabilitation is often characterized by prolonged duration and low adherence post-discharge, which significantly undermines the effectiveness of rehabilitation training (22).

Crucially, effective communication and coordination among the stroke recovery team, encompassing the patient, family, caregivers, medical professionals, psychologists, and social workers, are fundamental to maximizing the efficacy of recovery efforts (22). The absence of such collaboration can severely limit the potential of rehabilitation in stroke survivors. Future research is therefore focused on actively enhancing patients' self-management abilities and improving the outcomes of rehabilitation.

The array of studies conducted on atherosclerosis substantially enriches our comprehension of the disease's pathogenesis and treatment modalities. They shed light on the mechanisms driving atherosclerosis-related conditions and offer guidance to policymakers and healthcare practitioners in addressing the escalating global burden of these diseases. Moreover, these studies provide fresh perspectives for the creation of innovative pharmacotherapies and therapeutic strategies. A key aspect of this research is the clarification of the molecular underpinnings and clinical manifestations of atherosclerosis, which paves the way for more precise, individualized interventions and the practice of personalized medicine. This approach is crucial for enhancing patient care, especially as it aligns with evolving healthcare paradigms. Given the aging global population (26), effectively managing atherosclerosis—a complex and chronic condition—emerges as a significant challenge. The research in this field aims to address this challenge through the synergy of interdisciplinary collaboration and the translation of research findings into practical applications. This holistic approach is vital for advancing our ability to combat and manage atherosclerotic diseases more effectively, ultimately leading to improved health outcomes on a global scale.

## Author contributions

YX: Conceptualization, Methodology, Project administration, Resources, Writing – original draft. XZ: Conceptualization, Data curation, Formal analysis, Investigation, Resources, Writing – original draft. W-JT: Conceptualization, Methodology, Project administration, Resources, Revising – original draft.
